# The Management of Retinal Detachment: Techniques and Perspectives 2018

**DOI:** 10.1155/2019/8185619

**Published:** 2019-02-03

**Authors:** Elad Moisseiev, Anat Loewenstein, Ala Moshiri, Glenn Yiu

**Affiliations:** ^1^Department of Ophthalmology, Meir Medical Center, Affiliated to the Sackler School of Medicine, Tel Aviv University, Tel Aviv, Israel; ^2^Incumbent, Sidney Fox Chair of Ophthalmology, Department of Ophthalmology, Tel Aviv Medical Center, Affiliated to the Sackler School of Medicine, Tel Aviv University, Tel Aviv, Israel; ^3^Department of Ophthalmology & Vision Science, University of California Davis Eye Center, Sacramento, CA, USA

Retinal detachments are a common ocular condition that may have significant implication for patients' vision and wellbeing. They are frequently encountered by ophthalmologists of all subspecialties and possibly represent the quintessential challenge of vitreoretinal surgeons as these cases require early referral, thorough examination, a decision regarding the type of surgery, and properly timed intervention. Although surgical techniques have greatly improved over the years, surgery for retinal detachment repair remains technically challenging and requires skill, experience, and the proper instrumentation. About 60 years ago, retinal detachment was an incurable untreatable condition leading to irreversible vision loss, but today it is repairable in the vast majority of cases.

Advances in the understanding of retinal detachments and their treatment are continuously being made, with new instrumentation and techniques reported by many clinicians and researchers from all over the world. This annual special issue is intended to serve as a platform for sharing current data and new innovations in the management of retinal detachments.

In this issue, R. Kassem et al. performed a meta-analysis focusing on the timing of retinal detachment following cataract surgery. In their impressive study, which included more than 3 million eyes, the overall risk of retinal detachment following cataract surgery was found to be 1.16%, substantiating the known association between the two. The mean time for the occurrence of retinal detachment following cataract surgery was 1.5–2.3 years, and the authors suggest that proper timing of long-term follow-up after cataract surgery may assist in earlier detection of retinal detachment. The study also includes a comprehensive review of the literature on the association between cataract surgery and retinal detachment and the risk factors for this complication.

P. Kanclerz and A. Grzybowski contributed a thorough review on the use of gases in vitreoretinal surgery, covering their evolution, types, different characteristics, and alternatives. The review focuses on the possible complications of these gases, such as anterior chamber migration and endothelial damage, intraocular pressure elevation, cataract progression, and hypotony. This comprehensive review illuminates many of the important considerations that should be made when choosing and using gases in vitreoretinal surgery.

The use of 27-gauge systems for vitreoretinal surgery is gaining popularity, and two studies in this issue compared 27-gauge and 25-gauge vitrectomy for the repair of primary rhegmatogenous retinal detachment. D. Veritti et al. reported results of a prospective study including 74 eyes, and K. Otsuka et al. reported results of a retrospective study including 62 eyes, both with follow-up of over 6 months. In both studies, excellent results were achieved, with no difference in reattachment rates, visual improvement, or complications between the two systems. These results indicate the safety and efficacy of the 27-gauge instrumentation, which can be used for retinal detachment repair, and will likely increase in popularity in the near future.

Finally, V. Bonfiglio et al. reported an innovative technique for the repair of macula-on retinal detachments with intermediate breaks with vitreous traction. In this study, 32 such phakic eyes were treated by a limited 25-gauge vitrectomy, performed under air, releasing traction of the breaks and endolaser around them after the retina had reattached. All eyes were followed for 12 months, with excellent results comparable to those achieved by conventional vitrectomy. Importantly, none of the eyes demonstrated cataract progression, indicating this technique can be equally effective and safely used in select phakic patients.

